# Implementation of an insecticide-treated net subsidy scheme under a public-private partnership for malaria control in Tanzania – challenges in implementation

**DOI:** 10.1186/1475-2875-8-201

**Published:** 2009-08-21

**Authors:** Ritha JA Njau, Don de Savigny, Lucy Gilson, Eleuther Mwageni, Franklin W Mosha

**Affiliations:** 1 World Health Organization Country Office, P.O. Box 9292, Dar-es-Salaam, Tanzania; 2 Swiss Tropical Institute, Basel, Switzerland; 3 Centre for Health Policy, University of Witwatersrand, Johannesburg, South Africa; 4 Health Economics and Financing Programme, London School of Hygiene and Tropical Medicine, UK; 5 School of Public Health and Medicine, University of Cape Town, South Africa; 6 University of Lands, Dar-es-Salaam, Tanzania; 7 Tumaini University, Kilimanjaro Christian Medical College, Moshi, Tanzania

## Abstract

**Background:**

In the past decade there has been increasing visibility of malaria control efforts at the national and international levels. The factors that have enhanced this scenario are the availability of proven interventions such as artemisinin-based combination therapy, the wide scale use of insecticide-treated nets (ITNs) and a renewed emphasis in indoor residual house-spraying. Concurrently, there has been a window of opportunity of financial commitments from organizations such as the Global Fund for HIV/AIDS, Tuberculosis and Malaria (GFATM), the President's Malaria Initiative and the World Bank Booster programme.

**Methods:**

The case study uses the health policy analysis framework to analyse the implementation of a public-private partnership approach embarked upon by the government of Tanzania in malaria control – 'The Tanzania National Voucher Scheme'- and in this synthesis, emphasis is on the challenges faced by the scheme during the pre-implementation (2001 – 2004) and implementation phases (2004 – 2005). Qualitative research tools used include: document review, interview with key informants, stakeholder's analysis, force-field analysis, time line of events, policy characteristic analysis and focus group discussions. The study is also complemented by a cross-sectional survey, which was conducted at the Rufiji Health Demographic Surveillance Site, where a cohort of women of child-bearing age were followed up regarding access and use of ITNs.

**Results:**

The major challenges observed include: the re-introduction of taxes on mosquito nets and related products, procurement and tendering procedures in the implementation of the GFATM, and organizational arrangements and free delivery of mosquito nets through a Presidential initiative.

**Conclusion:**

The lessons gleaned from this synthesis include: (a) the consistency of the stakeholders with a common vision, was an important strength in overcoming obstacles, (b) senior politicians often steered the policy agenda when the policy in question was a 'crisis event', the stakes and the visibility were high, (c) national stakeholders in policy making have an advantage in strengthening alliances with international organizations, where the latter can become extremely influential in solving bottlenecks as the need arises, and (d) conflict can be turned into an opportunity, for example the Presidential initiative has inadvertently provided Tanzania with important lessons in the organization of 'catch-up' campaigns.

## Background

Malaria remains one of the major tropical challenges in the world today. Based on World Health Reports 1999–2004, the number of malaria deaths globally has been estimated at 1.1–1.3 million [1]. The most recent World Health report estimates that malaria incidence rates are 350–500 million per annum [2]. In the past three decades, malaria has, however, encroached upon areas where it had formerly been eradicated or had successfully been controlled, offsetting the gains attained in the latter half of the past century [3]. The disease is endemic in 107 countries with some 3.2 billion lives at risk of transmission. About 60% of the cases of malaria worldwide, 75% of global falciparum malaria cases and more than 80% of malaria deaths occur in Africa South of the Sahara. *Plasmodium falciparum *causes the vast majority of infections in this region and about 18% of deaths in children under five years of age [2].

Studies focusing on under-five children in African populations conclude that 600,000 children contract cerebral malaria yearly, with a case fatality rate of 20%. Severe malaria due to anaemia occurs in 1.5 – 6.0 million African children, with a case fatality rate of 15% [4]. Malaria is also a major threat to pregnant women and adversely affects foetal growth and newborn survival through low birth weight.

The socio-economic impact of malaria is extremely high in endemic countries. It has been observed that over the past 25 years the economic growth in malarial countries has been hampered [5]. It incapacitates the workforce, leading to decreased economic productivity and output in various sectors of the economy [6].

In the past decade, proven interventions, such as artemisinin-based combination therapy (ACT), the wide-scale use of insecticide-treated nets (ITNs) and a renewed emphasis in indoor residual house-spraying (IRS), are available for combating the disease. The challenge is to ensure that these relatively inexpensive interventions reach a major proportion of the population through universal coverage.

A number of global initiatives have been developed leading to increased awareness of malaria such as the Roll Back Malaria (RBM) initiative spearheaded by the World Health Organization's Director General in 1998, followed up by the Abuja Declaration on Roll Back Malaria by African Heads of State [7], and most recently a statement made by the World Health Assembly [8]. In this statement, member states are urged to establish policies and operational plans to ensure that at least 80% of those at risk of or suffering from malaria benefit from major preventive and curative interventions in accordance with WHO technical recommendations, by 2010 so as to ensure a reduction in the burden of malaria of at least 50% by 2010 and by 75% by 2015.

The Abuja targets were revisited in 2006 where all the indicators were raised from 60% to 80% on the basis of current opportunities and increasing visibility of financial support from the global community such as the Global Fund to Fight AIDS, Tuberculosis and Malaria (GFATM), President's Malaria Initiative (PMI), World Bank Booster funding and the Bill and Melinda Gates Foundation [9].

During the last 15 years, a large body of evidence has been compiled on the key technical and managerial aspects of ITNs as well as on their efficacy and effectiveness. A systematic review of 22 randomized controlled trials showed a consistently high impact of ITNs [10,11] preventing malaria episodes. Current debates around ITNs, internationally and nationally where malaria is endemic, is the appropriate delivery mechanisms for wide-scale use of ITNs [12-15]. The advantages and challenges of the various delivery mechanisms have been discussed.

Tanzania has been on the forefront in the promotion of the use of ITNs from research projects in the 1980s and 1990s [16-18] and validation in larger field trials in early 2000 [19,20] to a nation-wide programme through a discount voucher scheme delivered through a public-private partnership approach [21,22]. The ITN activities in Tanzania were closely linked to international developments including: randomized controlled trials [23-25], technological developments in the home treatment kits [26], rationalization of taxes and tariffs and expansion, and investment in Long-Lasting Insecticide-treated Nets (LLIN).

Through the nationwide discount voucher scheme, pregnant women access the vouchers at the Antenatal clinics in public health facilities. The voucher is redeemed for a mosquito net of her choice at a shop in the catchment area of the health facility by adding a variable top-up, ranging between 0.75 – 1.25 USD depending on the size of the mosquito net purchased [21,22].

Since April 2008, the pregnant woman voucher has been complemented with an infant voucher scheme, which uses the same delivery mechanism as the pregnant woman voucher through the Antenatal Clinics. It is also a country-wide initiative. Until mid-year 2008, the two schemes combined totaled a redemption of 3.5 million nets as a share of the mosquito nets available in the country through these groups [27].

A recent study carried out in a rural setting in Tanzania showed that a combination of interventions, such as vouchers for the vulnerable groups, free mosquito nets and full-cost nets in the local market have proved to raise the coverage of mosquito nets in different segments of the population substantially thus complementing each other [28]. A lot of research on ITNs has concentrated on the outcomes and impact of the intervention on malaria and most recently investigations have been done on various delivery mechanisms to ensure the tool is available to the target groups as alluded to above. However, there is very little covered in the research in terms of health policy analysis in malaria [29] and Njau et al, *in press*). However health policy analysis has been used in HIV/AIDS and has also been used to describe health sector reforms in the country [30-33].

Community health financing (CHF) was introduced in Tanzania through the health sector reforms since 1995 [31,34], with an overarching aim of resource generation and equity. Using the health policy analysis framework, the authors point to the top-down approach in the development of the community health financing policy, where there was no interaction of the central Ministry of Health (MoH) with the sub-national levels in the conceptualization of the CHF[30,31]. Furthermore managerial weaknesses were also observed. These included lack of supervision and accountability by the district managers.

In Mwanza, Tanzania, the health policy analytical framework was used to describe extensively how HIV/AIDS research findings were successfully been translated to policy [32]. The trial demonstrated the effectiveness of improved treatment services for Sexually Transmitted Infections in preventing HIV infections. The factors that influenced the policy impact of the research results included: 1) research data packaged in a simple manner digestible by politicians, 2) researchers and policy makers formed strategic alliances for the policy shift, and 3) the policy environment was conducive.

### Methodological approach

**Figure 1:**
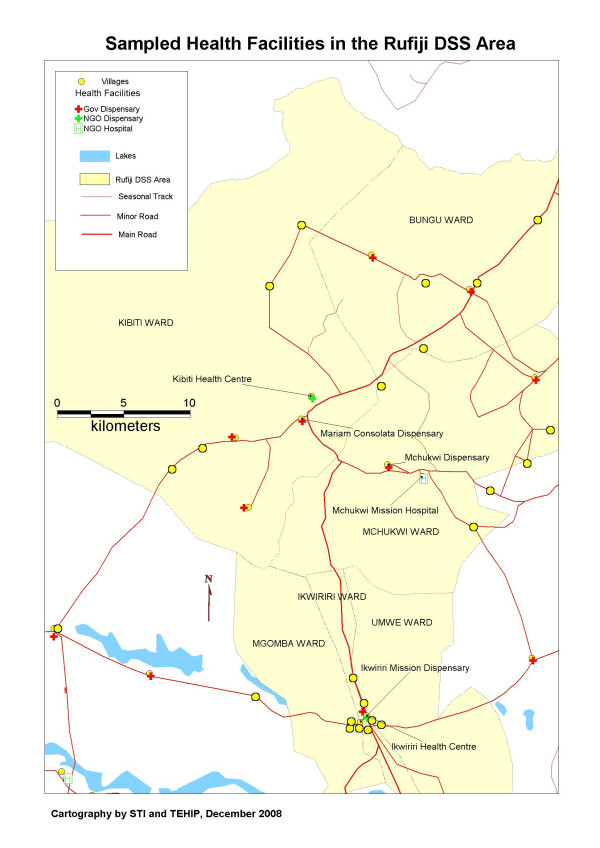
**Sampled health facilities and wards in the Rufiji Demographic Surveillance Site**.

The present research uses the health policy analysis framework [35] to analyse the implementation of a unique model embarked upon by the government of Tanzania – 'The Tanzania National Voucher Scheme (TNVS)' – in the control of malaria. The present study concentrates on the challenges faced by the scheme in the public-private partnership (PPP) approach during the key pre-implementation phase (2001 – 2004) and implementation phases (2004 – 2006). The process of decision-making and how the discount voucher scheme was conceptualized is described elsewhere (Njau *et al*, in press). A case study approach is used to describe in-depth the challenges that the stakeholders' faced in the two phases mentioned in this synthesis and how they attempted to overcome them [24]. The analysis unpacks the different challenges encountered at the international level, including issues confronted in signing the GFATM grant proposal, which supported the implementation of the scheme, and, at the national level, the marketing challenges in promoting the ITNs. Lastly, observations were made at the community level on the challenges encountered by the Council Health Management Teams (CHMTs) and health care providers in the context of a cross-sectional survey conducted at the Rufiji Demographic Surveillance Site (DSS) (Figure 1).

### Data collection tools

Qualitative and quantitative methods were used in the study to highlight the challenges experienced at the different levels of the health care delivery system in the implementation of the country-wide subsidy scheme.

### Qualitative methods

The qualitative methods included: key informant interviews from the national and district levels, policy characteristic analysis, stakeholders' analysis and focus group discussions.

#### Key informant interviews

The interviews were carried out through 2005 and 2006. A balanced selection of key informants were interviewed from the MoH, the National Malaria Control Programme (NMCP), the CHMT in Rufiji district, Reproductive and Child Health (RCH) coordinators in randomly selected health facilities in Rufiji, stakeholders sub-contracted by the MOH to implement the TNVS and stakeholders involved in malaria control through technical support to the MOH. Other groups interviewed included influential people from the community and the private sector. The private-for-profit sector included some of the Tanzania Net Manufacturing companies, Price Waterhouse Coopers, wholesalers and retailers while the private-not-for-profit included Population Services International (PSI) and the Christian Social Services Commission (CSSC). The synthesis identified 14 key informants from the onset to be interviewed, however, 10 more were added through a snowball effect, as key people mentioned through the first interviews were sought and added to the list [36].

The review involved going through relevant documents related to the socio-economic and political environment of the country, such as the National strategic plans, the health reform polices and specifically the national malaria control plans. Published data on malaria control and public health policy were extensively reviewed, while secondary data included the NMCP annual reports, TNVS reports, CHMT reports and excerpts from the Tanzania National Coordinating Mechanism. The document review enabled the analyst to gather a historical perspective of the events leading to the development and implementation of a PPP initiative for scaling up ITNs (Njau *et al *in press). The time-line of events, which was derived from the document review, identifies the key steps in the implementation of the scaling-up of ITNs country-wide in a chronological manner (Tables 1 and 2).

**Table 1:** Time line of events in the implementation of an insecticide treated nets subsidy scheme under a Public – Private -Partnership for malaria control in Tanzania

Year	Key National Factors	Key Global Factors
1984 – 1994	Research in Tanzania on insecticide treated nets in experimental huts and small-scale field trials	
1994	Kunduchi Meeting, Dar-es- Salaam on 'Net Gain'* – first international/broad discussion on social marketing of ITNs	
1996	British Council Meeting, Dar-es-Salaam Tanzania Essential Health Intervention Project (TEHIP) invites stakeholders to discuss on 'How to measure mortality impact in populations,' prior to the launch of the Demographic Surveillance System in Rufiji. The KINET project was conceptualised here	
1994 – 1996		Randomized controlled trials on the impact of ITNs on overall childhood mortality
1996	DRC – Development of home-treatment kit for the treatment of mosquito nets	
1997	International Advisor from the Tanzania Essential Health Project and Director of Promotional Services International approach A to Z Textile Mills on the issue of increasing production on mosquito nets (This was during the International Public Health Association meeting in Arusha).	First International Conference on Bed-nets and other treated materials for the prevention of malaria**, Washington, USA
	The Tanzania Essential Health Intervention Project (TEHIP) is launched in Rufiji and Morogoro Rural	
1998	Meeting at White Sands Hotel, Dar-es-Salaam to discuss Marketing Challenges in the Promotion of ITNs***. This was a multisectoral meeting involving both the public and private and the NGO sector. Major agenda was the rationalization of taxes and tariffs on ITNs in Tanzania	The Roll Back Malaria Partnership is created
1998 – 2001	First and second phase of social marketing of Insecticide treated nets – Population Services International	
1999	Three textile manufacturing companies scale up production of mosquito nets [A to Z Textile Mills and Sunflag Textile industries in Arusha and Tanzania Textile Manufacturing Limited (TMTL) in Dar-es-Salaam]	Second International conference on Insecticide treated Nets for the prevention of malaria, Dar-es-Salaam****
	Ad hoc meeting of a decides to go to national scale	
	Multilateral Initiative for Malaria (MIM) – Dar-es-Salaam	
1999/2000	Rationalization of Taxes and tariffs on mosquito nets. Legislation is passed mosquito nets are zero rated VAT and import duty. Mosquito nets are classified under the essential drug list. Polyester yarn is still levied 20% VAT but is refundable to the manufacturer on production of relevant documents	
2000/2001	Insecticides for treating mosquito nets are zero rated VAT and import duty, polyester yarn is also zero rated VAT and import duty	

* First International conference on bednets and other treated materials for the prevention of malaria, USAID Report, 1997

*** NMCP/IDRC/PATH Marketing challenges for the public and private sector, white Sands Hotel, Dar-es-Salaam, 1998

**** Insecticide treated nets in the 21^st ^Century, Report on the Second International conference on ITNs, Dar-es-Salaam, Tanzania, 1999

**Table 2:** Time line of events in the implementation of an insecticide-treated nets subsidy scheme under a public-private partnership for malaria control in Tanzania (period from 2000 to 2006)

Year	Key National Factors	Key Global Factors
2000	Finalization of large scale social marketing programme on ITNs in South-East Tanzania (Abdullah et al 2000; Schellenberg et al 2001)	
	Meeting with stakeholders on 'Going to Scale with Insecticide Treated Nets' in Tanzania^ł^	
	A strategic plan on scaling up of ITNs in Tanzania is developed^łł^	
	Multisectoral task force is formed to spearhead the implementation of ITNs in the country	
	Speech by the His Excellency the President of the United Republic of Tanzania; President William Mkapa at a fund raising dinner for the comprehensive control of malaria in Zanzibar	
2001	Terms of reference for ITN Steering Committee and Consultative group developed Task force is therefore converted to an 'ITN Steering Committee' chaired by the Chief Medical Officer of the MOH	
2002–2003	Third phase of social marketing of Insecticide treated nets (SMARTNET) – Population Services International	
2002	An implementation plan for scaling up of ITNs in Tanzania is developed	GFATM awards Tanzania 19.8 million USD for the NATNETs project for a period of 3 years
	All locally produced mosquito nets are bundled with an insecticide re-treatment kit	
	Submission of a proposal on the National Insecticide Treated Nets implementation (NATNETs) plan support to the GFATM Round 1	
	Reintroduction of VAT on raw materials for the production of mosquito nets by the MOF	
2003	ITN team leader recruited at the beginning of year and Administrator and Communication Officer recruited at the end of the year. Financial support towards the technical staff for the ITN cell made available through SDC. Proposal prepared by STI^łłł ^and NMCP	International community advocate for free nets
	Steering Committee reconstituted, reflecting a much more narrower committee	
	Development Cooperation Ireland contribute financially towards the NATNETs programme	
	Sensitization of members of Parliament on the disease burden of malaria, its impact on the economy and strategies for its prevention with emphasis on the use of ITNs	
2004	Implementation of the NATNETs project delays until October 2004 – launch by Honourable Vice President	
	Districts officially informed to examine ways in which existing ITN activities (revolving fund, sale of nets by health facilities and free distribution of ITNs) can be phased out once the TNVS is introduced in respective districts	
	Price WaterHouse Coopers is appointed as the Local Fund Agent for the NATNET project	
	TNVS Advocacy Meeting to Regions in the First Roll out Phase and other stakeholders, Morogoro	
	Launching of LLIN produced by A to Z by the Honourable President of the United Republic, Benjamin Mkapa	
2004 – 2005	An additional net manufacturing company becomes operational established in Northern Tanzania; Motex Textiles Ltd	
2005	SDC supports second phase of the ITN cell up to 2008. Proposal prepared by STI and NMCP	World Economic Forum, Davos, Switzerland – Honurable former Head of State – Benjamin Mkapa receives a donation towards malaria control in Tanzania
	Monitoring and Evaluation of the TNVS by IHRDC and LSHTM – 2005 and 2006	
	The United Republic of Tanzania invests in scale-up of Long Lasting Insecticide Treated Nets in the A to Z Textile Manufacturing company in Arusha through building a road to the new manufacturing site and provision of water	

^ł ^PricewatersHouseCoopers and Population Service International, Going to scale with insecticide treated nets, Dar-es-Salaam, March 2000

^łł ^United Republic of Tanzania, Ministry of Health, National Malaria Control Programme, Taking insecticide treated nets to national scale in Tanzania, November, 2000

#### Stakeholders' analysis

Stakeholders' analysis was also used in the research and enabled the reviewer to understand the actors' behaviour, interests, intentions and interactions. It further allowed the assessment of the actors influence and resources that they brought to bear on decision making in the development and the implementation of a PPP initiative in the wide-scale use of ITNs [36]. The information for each group/actor was arrayed according to the group interests, the level of resources the group or actor possessed and the groups' position on the PPP initiative. The resources that were identified in the stakeholders analysis were drawn from the preliminary policy characteristic analysis [37,38]. These included (a) technical resources, described here as the inherent technical expertise that the stakeholder has that would be critical to the implementation of the policy or by virtue of the stakeholders position in relation to the implementation of the policy (for example, the presence of the Tanzania Net Manufacturers in the country providing a market for mosquito nets); (b) political resources, expounded here as the influence that the stakeholder has on the ITN subsidy scheme through direct or indirect interaction with government officials; and (c) financial resources, the stakeholders influence over the economic situation. For example, PSI conducting social marketing of ITNs in the remotest parts of the country, or the UK Department for International Development availing financial support towards social marketing initiatives.

The stakeholders' analysis enabled the synthesis to look retrospectively at the key actors and groups of actors and their influence, or lack of it, in the development and the implementation of a PPP in scaling-up ITNs country-wide. The outcome enabled the analyst to draw recommendations for future health policies in relation to optimizing participation under conditions of limited resources [39].

#### Force-field analysis

Force-field analysis and micro-political mapping were used in support of the stakeholders' analysis, where the former clarified the array of support on the discount voucher scheme by different stakeholders and micro-political mapping showed the sources of actors influence over the implementation of the ITN subsidy scheme in the decision making phase, the pre-implementation and implementation phases [40].

#### Focus group discussions

Two focus group discussions (FGDs) were conducted where purposive sampling was used to select individuals from two separate villages from Ikwiriri and Kibiti divisions under the Rufiji DSS. Each group, consisting of 8 – 12 individuals, included women of child-bearing age and some women in these groups were pregnant and others had infants at the time of the survey. Older women in the society of different socio-economic status were also included [41]. Each FGD took between 45 – 50 minutes. Guiding questions were prepared with the major aim of finding out from the women what the major health problems in the village were, how they addressed them and whether they were acquainted to the discount voucher scheme.

### Qualitative data analysis

#### Triangulation

Triangulation of the information accrued from the interviews was done through published and grey literature.

#### Policy characteristic analysis

A policy characteristic analysis was used to provide a systematic understanding of what the policy change was designed to do, the context in which the policy was implemented, the consequences of the change and the reactions of the public and/or the bureaucracy [37].

#### Grounded theory

A number of informants came-up with quite similar issues and perspectives on key subjects related to the issue. One or two quotes representing the thoughts are quoted to reflect the thoughts of the key actors; however they are kept anonymous. Controversial issues and perceptions of some key actors were also quoted to enlighten the reader on the hurdles that the implementers faced and how they were able to overcome them. In most instances the quotations are validated through the literature review which was conducted. This approach is described as the grounded theory [42,43].

### Quantitative analysis

A retrospective cohort of women of child bearing age from Ikwiriri and Kibiti divisions of the Rufiji district in the Coast Region – the Rufiji DSS database was followed-up twice in the survey coinciding with the normal DSS updates (Figure 1). The first follow-up was during the February 2005 – May 2005 cycle, immediately after the discount voucher was launched in the district. The second follow-up was in the June 2005 – September 2005 cycle, when the discount voucher scheme had been operational for eight months. The Rufiji DSS involves continuous surveillance of households and members within households in cycles or intervals of four months each. The information collected in these cycles includes demographic, household, socioeconomic and environmental characteristics of the population [44].

The sample size estimation for the number of households with the probability of having pregnant women was calculated using the estimates of the proportion of pregnant women and women of child bearing age in the RDSS census of 2003. The anticipated proportion of pregnant women with a mosquito net through the TNVS was estimated at 50%. With a precision of 5% and an average household of 4.7, a total of 1048 households were sampled. Systematic random sampling was used, where nine villages were selected from Kibiti, Mchukwi and Bungu wards of Kibiti division, and three villages from the Ikwiriri, Mgomba and Umwe wards (Figure 1).

A semi-structured questionnaire was used to collect information on ITN possession and use. This was triangulated with subsequent surveys carried out by the monitoring and evaluation contractors during the implementation of the TNVS [45]. The questions included knowledge of TNVS, access to ITNs, barriers to access and redemption mechanisms. The relationship between socio-economic status and health has been an area of increasing interest in recent years [46,47]. A number of studies have used the socio-economic status to predict variance against mortality of children under five (infant and <5 mortality) and health interventions such as household possession of mosquito nets [44,48,49]. Each member of the cohort of women of child-bearing age was matched with the respective socio-economic household indicator index derived from the Rufiji DSS in 2000 for mosquito net possession. The socio-economic data was collected during the October 2000 – January 2001 Rufiji DSS cycle. This included asset ownership, housing conditions, source of energy for cooking, type of water and sanitation [44]. Principle Component Analysis, using Stata 7.0 (Stata Corporation), was applied to the socio-economic data to obtain a proxy index for household socio-economic status [44,50]. The cohort was divided into socio-economic quintiles for analyses [44].

### Data management for the quantitative analysis

Data was entered into Excel^® ^and processed in Stata Version 9.0^®^. Data was entered twice and cleaned by data managers at the Rufiji DSS. The analysis was done for computing frequencies, Chi square tests and calculating Confidence intervals for examining the real differences in net use amongst the cohort of women of child-bearing age followed up and the difference in net acquisition of the different social economic groups.

### Challenges in the implementation of the country wide subsidy scheme through a PPP approach

The first part of this synthesis analysed the period of 'building momentum' toward a country-wide scale-up of a discount voucher scheme. The main conclusion was that the PPP approach to the initiative was made through incremental approaches both at the national and international levels and the process was dynamic and complex and far from linear. Secondly, the analysis also observed that the policy implementation has stretched over several years and is still evolving.

Tanzania is a fairly stable country politically with a unitary system of government embedded in its socialist history [51]. Since 1995, the government has turned to a market-based economy and has in the recent past introduced a series of comprehensive frameworks to address the issues surrounding poverty, with Vision 2025 as the overarching development framework [51]. In this relatively stable context and a maturing open market, the voucher scheme for the control of malaria was not implemented without challenges and 'hiccups.'

**Figure 2:**
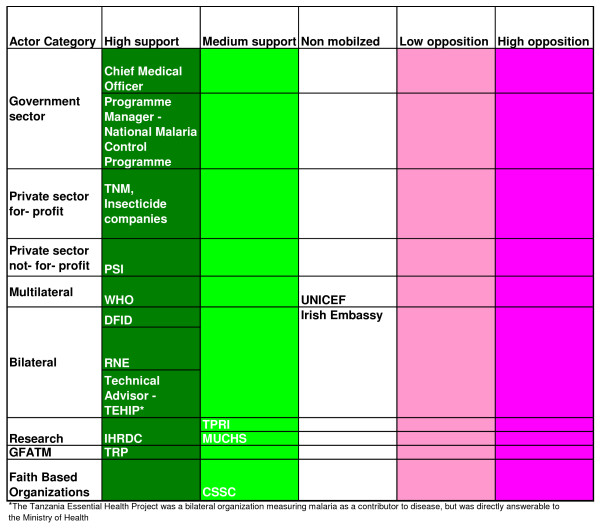
**The process of decision-making – Actors position in the process of adopting a public-private-partnership approach to an ITN subsidy scheme- 1998 – 2001**. **Notes**: Figures 2, 3 and 4 indicate the degree of support or opposition in relation to the process of adopting a public-private-partnership approach to an ITN subsidy scheme in different implementation phases; Phase I – 1998 – 2001; Phase II – 2001 – 2004 and phase III – 2004–2006. It also shows how the actors position shift over time (Varvasvsky and Brugha, 2000). The source of information includes a stakeholder's analysis and documentary data.

**Figure 3:**
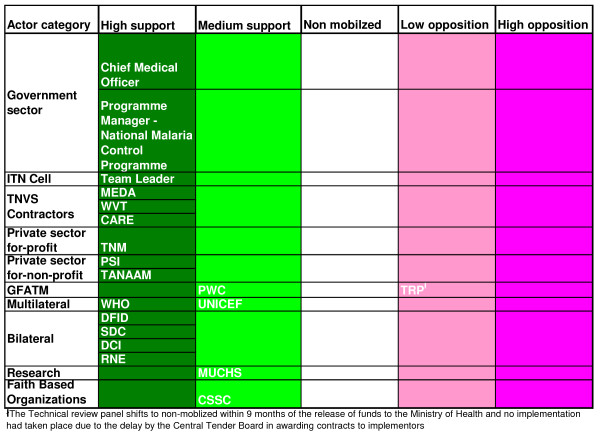
**Key steps towards implementation – Actors position in the steps towards implementation of a public – private – partnership approach to an ITN subsidy scheme – 2001 – 2004**.

**Figure 4:**
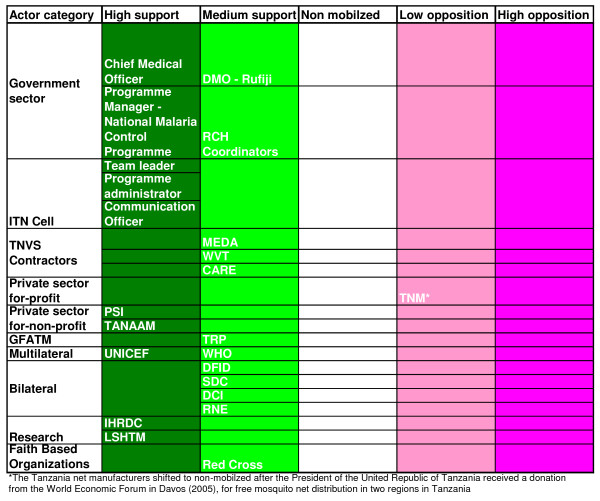
**Implementation phase – Actors position in the implementation of a public – private – partnership approach to an ITN subsidy scheme – 2004 – 2005**.

**Figure 5:**
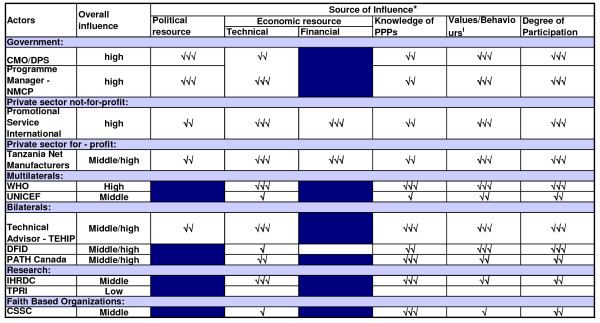
**The process of decision making – The source of actors influence over the implementation of an ITN subsidy scheme – 1998 – 2001**. Source of influence figures adapted from; Gilson L., Doherrty J., Lake S., M^c^intyre D., Mwikisa C and Thomas S.2003. The SAZA study: implementing health financing reform in South Africa and Zambia. Hlth Policy and Planning. 18 (1): 31–46. Political resource – described here as the influence that the stakeholder has on the ITN subsidy scheme through direct or indirect interaction with government officials. Government here is denoted as 'public institutions in which collective decisions are made into laws that affect the whole society; parliament, the executive, the bureaucracy, ministries or departments of state (Walt, 1994)'. Technical resource – described here as the inherent technical expertise that the stakeholder has that would be critical to the implementation of the policy or by virtue of the stakeholders position in relation to the implementation of the policy. For example the presence of the Tanzania Net Manufacturers in the country providing a market for mosquito nets. Financial resource – the stakeholders influence over the economic situation. For example, Promotional Service International conducting social marketing of ITNs in the remotest parts of the country, or DFID availing financial support towards social marketing initiatives. Number of ticks indicates relative level of influence derived from source: 3 ticks = high influence; no tick = no influence. For example the influence of the Chief Medical Officer derived from technical source was due to his authoritative position as the lead advisor in health within the Ministry of Health and as a Chairperson of the steering committee of the Tanzania National Voucher Scheme. While the influence of the Tanzania Net Manufacturers derived from technical source is their expertise in the local production of mosquito nets, hence the availability of the public health commodity within the country locally. Values/behaviours refers to the differing behaviours of the actors (e.g. rooted in clear principles or values, tactical and strategic, expressing commitment). For example the CSSC was highly committed to PPPs as a general health policy and was spearheading it so that it could be institutionalized; however it perceived that the ITN subsidy scheme had not adequately involved Faith Based Organizations at the grass root level

**Figure 6:**
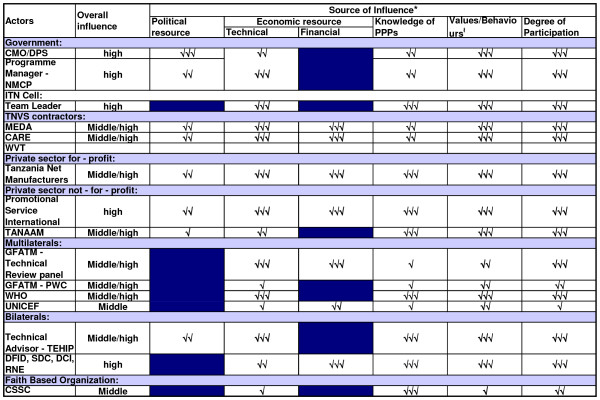
**Key steps towards implementation – The source of actors influence over the implementation of an ITN subsidy scheme – 2001 – 2004**. Source of influence figures adapted from; Gilson L., Doherrty J., Lake S., M^c^intyre D., Mwikisa C and Thomas S.2003. The SAZA study: implementing health financing reform in South Africa and Zambia. Hlth Policy and Planning. 18 (1): 31–46. Political resource – described here as the influence that the stakeholder has on the ITN subsidy scheme through direct or indirect interaction with government officials. Government here is denoted as 'public institutions in which collective decisions are made into laws that affect the whole society; parliament, the executive, the bureaucracy, ministries or departments of state (Walt, 1994)'. Technical resource – described here as the inherent technical expertise that the stakeholder has that would be critical to the implementation of the policy or by virtue of the stakeholders position in relation to the implementation of the policy. For example the presence of the Tanzania Net Manufacturers in the country providing a market for mosquito nets. Financial resource – the stakeholders influence over the economic situation. For example, Promotional Service International conducting social marketing of ITNs in the remotest parts of the country, or DFID availing financial support towards social marketing initiatives. Number of ticks indicates relative level of influence derived from source: 3 ticks = high influence; no tick = no influence. For example the influence of the Chief Medical Officer derived from technical source was due to his authoritative position as the lead advisor in health within the Ministry of Health and as a Chairperson of the steering committee of the Tanzania National Voucher Scheme. While the influence of the Tanzania Net Manufacturers derived from technical source is their expertise in the local production of mosquito nets, hence the availability of the public health commodity within the country locally. Values/behaviours refers to the differing behaviours of the actors (e.g. rooted in clear principles or values, tactical and strategic, expressing commitment). For example the CSSC was highly committed to PPPs as a general health policy and was spearheading it so that it could be institutionalized; however it perceived that the ITN subsidy scheme had not adequately involved Faith Based Organizations at the grass root level

**Figure 7:**
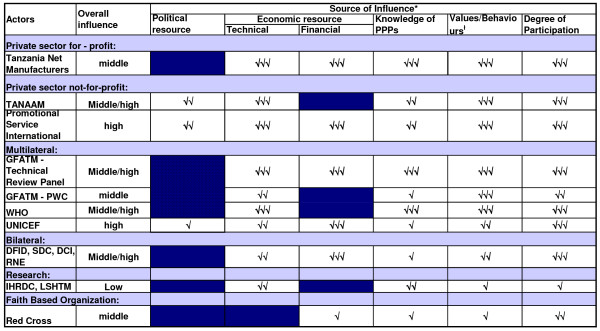
**Implementation phase – The sources of actors influence over the implementation of an ITN subsidy scheme – 2004 – 2005**. Source of influence figures adapted from; Gilson L., Doherrty J., Lake S., M^c^intyre D., Mwikisa C and Thomas S.2003. The SAZA study: implementing health financing reform in South Africa and Zambia. Hlth Policy and Planning. 18 (1): 31–46. Political resource – described here as the influence that the stakeholder has on the ITN subsidy scheme through direct or indirect interaction with government officials. Government here is denoted as 'public institutions in which collective decisions are made into laws that affect the whole society; parliament, the executive, the bureaucracy, ministries or departments of state (Walt, 1994)'. Technical resource – described here as the inherent technical expertise that the stakeholder has that would be critical to the implementation of the policy or by virtue of the stakeholders position in relation to the implementation of the policy. For example the presence of the Tanzania Net Manufacturers in the country providing a market for mosquito nets. Financial resource – the stakeholders influence over the economic situation. For example, Promotional Service International conducting social marketing of ITNs in the remotest parts of the country, or DFID availing financial support towards social marketing initiatives. Number of ticks indicates relative level of influence derived from source: 3 ticks = high influence; no tick = no influence. For example the influence of the Chief Medical Officer derived from technical source was due to his authoritative position as the lead advisor in health within the Ministry of Health and as a Chairperson of the steering committee of the Tanzania National Voucher Scheme. While the influence of the Tanzania Net Manufacturers derived from technical source is their expertise in the local production of mosquito nets, hence the availability of the public health commodity within the country locally. Values/behaviours refers to the differing behaviours of the actors (e.g. rooted in clear principles or values, tactical and strategic, expressing commitment). For example the CSSC was highly committed to PPPs as a general health policy and was spearheading it so that it could be institutionalized; however it perceived that the ITN subsidy scheme had not adequately involved Faith Based Organizations at the grass root level

Coational shifts in support or opposition of the scheme was observed in the implementation phase as the major stakeholders 'increased' or 'decreased' their interest in the subsidy programme. The stakeholders' analysis vividly brought forward the attributes that the individual actors had in influencing the policy depending on the strength or the power that they possessed. Figures 2, 3 and 4 highlight the actors' position in the process of decision-making, key steps towards implementation and the implementation phase, respectively. Figures 5, 6 and 7 stipulate the sources of actors influence over the implementation of the subsidy scheme in all three phases.

### Marketing challenges in promoting ITNs – White Sands meeting 1998

As the agenda had been set, with a firm foundation for the PPP approach in scaling up ITNs, a number of challenges were to be addressed in the pre-implementation stage. One of the milestones in the momentum that was being created in providing a conducive environment for the up-scaling of ITNs was a meeting held at the White Sands Hotel in 1998 [52]. A serious barrier to ITN promotion identified at this meeting was taxation. The meeting outlined plans of action for follow-up and assigned various tasks to the different stakeholders. Some of the tasks included outlining a strategy to petition against the introduction of Value Added Tax (VAT) on mosquito nets and insecticides, lobbying members of parliament and influential individuals. Wide dissemination of the meeting recommendations to key stakeholders such as the World Bank (both nationally and internationally), WHO, the World Trade Organization and the media. Finally, in the MOF Bill of 1999/2000, the tireless efforts of all stakeholders proved fruitful when mosquito nets were zero rated for import duty and VAT, and classified under the essential drug list of the MoH. The 20% VAT levied on 100% polyester yarn was refundable to the manufacturing companies as rebate on production of relevant documents, which had been audited and certified by the Tanzania Revenue Authority. In this manner, they established the volume of imported yarn, which was actually used to produce a certain number of mosquito nets (as polyester yarn could also be used to make other fabrics) [53].

### A revisit of the rationalization of taxes and tariffs on ITNs

An issue already well dealt with during the decision making stage, appeared once again in the pre-implementation phase (phase II): the issue of taxes and tariffs related to ITNs, with the re-introduction of VAT by the Ministry of Finance (MoF) in May 2002 on raw materials for production of mosquito nets. This was discussed at length at the steering committee of the National ITN Strategy (NATNET) in 2003. One of the members of the committee remarked as follows;

"*Lobbying is needed to re-instate zero rating on VAT as well as zero rating customs duty. There is a risk if this is not achieved in this budget (as the fiscal year for the Government begins June/July – hence any amendment had to be done before that) there will never be a revision. The Minister of Health has been made aware of this issue and information has been sent to the Ministry of Finance. The current tax policy favours imported nets as finished products imported are not subject to VAT. It was noted however that imported nets were not bundled and due to the uneven playing field of the imported nets versus locally produced nets there was a general supply shortfall of mosquito nets in the country (Steering committee member, 7*^*th *^*March 2003)*."

At the time of this synthesis the members of the NATNET steering committee were the Chief Medical Officer (the chairperson), the Programme Manager of the NMCP (the secretariat), the ITN Cell Team Leader, the UK Department for International Development, the Swiss Development Corporation, the President's Malaria Initiative, the Irish AID, WHO, UNICEF and PSI.

#### A tax reform task force is set-up

The NATNETs steering committee set-up a 'Tax reform task force,' with the objective of (a) providing an assessment of the changes at that particular time in the VAT legislation on the importation and local manufacture of mosquito nets, and (b) to make recommendations as to how to minimize the effect of the changes such that the tax burden was removed from the local manufacturer of mosquito nets.

On completion of its assignment, the task force observed that the amendments to the new taxes effectively restored the position of the old VAT Act of July 1998, where the mosquito nets produced locally and all the raw materials and overheads were VAT exempt. They were not able to claim back any VAT to the extent that they relate to domestic sales. In this case they would have to absorb this irrecoverable VAT by accepting reduced margins or passing it over to the consumer, through an increase in their selling price with the risk of reducing sales. However, importers of nets were zero rated for VAT at the point of entry. Thus, an uneven playing field between the locally-produced nets and the imported nets was created. The task force looked at the rationale behind the 2002 changes and summarized their recommendations as follows: the pressure for change in relief afforded in any country could be because of special interest groups who felt that the special relieves are disadvantaged to them or does not cater for their particular needs. Secondly, there may be pressure from within the government administration to change the reliefs available by increasing or decreasing them to remove inequalities of treatment, to remove ambiguities, to raise revenue or to give relief for social reasons[54].

The task force visited two of the three net manufacturing companies in the country at the time. The companies stated there would be an increase of 10 to 20% in mosquito net price should the government re-introduce VAT. The course of action suggested by the task force was that, a written submission had to be prepared by the MoH, from the senior health officials to the MoF, making a case for the changes required and proposals for the re-wording of the schedule. Subsequent to this submission, meetings were to be held between senior MOH officials and the Commissioner of Finance. The task force advised the MoH to request for the following in their submission to the MoF [54]: (a) to leave mosquito nets as VAT exempt, which implies no VAT at point of sale to consumer but VAT charged and not recoverable on all manufacturing inputs; (b) to levy a customs duty of 10% on ready-made mosquito nets. Therefore, leveling the playing field between imported nets and domestic mosquito nets.

#### Discourse between technical advisor and the TNVS steering committee

The NATNET steering committee did not agree with the recommendations of the task force and, therefore, wrote a memo to the Permanent Secretary of MoH [55], through the UK Department for International Development (as a member of the steering committee), requesting the restoration of zero rating on the sale of nets by domestic manufacturers and the removal of customs duty on material imported for the manufacture of mosquito nets. The memorandum also stressed that what the appointed task force had recommended on the tax issue was contrary to the international commendation made to Tanzania in early 2000 on the progressive rationalization of taxes and tariffs on mosquito nets and inputs in the production of mosquito nets and was a standing example to other malaria endemic countries.

To re-enforce the steering committees plea, the net manufacturers had written a memorandum to the Commissioner of VAT, dated 2^nd ^August 2002, requesting the Tanzania Revenue Authority to re-visit the new taxes imposed on domestic mosquito nets. The memorandum was copied to the Permanent Secretaries of the MoH, the MoF and the Ministry of Trade and Industry, the Programme Manager of the NMCP, WHO, the Tanzania Essential Health Intervention Project (TEHIP) and PSI.

#### Government medical stores department affected by tax reforms

Furthermore, the Medical Stores Department (MSD), which was the sole government semi-autonomous organization responsible for ordering pharmaceuticals, medical equipment and supplies on behalf of the government, found that the new tax amendment also affected them. They stated in their memorandum [56] which they addressed to the Permanent Secretary, MoH, *"......as a result 10% import duty is levied on all pharmaceuticals, including vaccines, TB and Leprosy drugs procured by her departments, and those given as donations. This has a very negative impact over our budget and ultimately to the end user, which is the Government health facilities. MSD has applied for a waiver of such taxes through the Permanent Secretary, Ministry of Finance (vide memorandum MSD/003/215/2002 dated 24^*th *^July 2002) and while waiting for an official response, more consignments are arriving at the sea and airports, demanding huge payment arising from taxes, port storage and demurrage."*

The MSD ended their memorandum by requesting the Permanent Secretary, MoH to discuss this issue bilaterally with the Permanent Secretary, MoF, as a matter of urgency. In a subsequent meeting of the NATNET steering committee on 25^th ^June 2003, it was observed that, as a result of pressure by the NMCP through the ITN Cell and other stakeholders (including private and public), the MoF endorsed in the 2004 Budget that VAT on mosquito nets was zero-rated and the customs duty on the yarn was also removed. This was a remarkable breakthrough in ensuring that the price of nets remained stable in the country.

The scenario depicted here reinforces the high administrative and technical resources which the individuals in the steering committee brought to bear in this particular high stake issue and its consequences in the public arena. The malaria agenda turned from 'business-as-usual' to a 'crisis' event. Without these concerted efforts the government would not have resolved the tax rationalization in a timely manner, which would have been detrimental to the PPP approach to scaling up ITNs in the country.

The members of the steering committee were consistently supporting the ITN subsidy scheme with high influence over the events that occurred in both the decision making stage and the pre- implementation phase (Figures 5 and 6) and high support in the decision making process (Figure 2).

### Challenges faced in signing the GFATM award

As the opinion leaders were being groomed such that there was heightened political commitment towards malaria control in the country; behind the scenes there were a number of teething problems in the pre-implementation phase with regards to the signing of the GFATM award.

The United Republic of Tanzania (URT), through its MoH, had been awarded USD 19 million from the GFATM for the implementation of the TNVS [57]. There were a number of critical and fundamental organizational arrangements, which the MoH and other government sectors had to lay down from the period that the GFATM grant agreement had been signed in 2002 to the launching of the Tanzania National Voucher Scheme in October 2004. It was also observed that in parallel, the GFATM, in these very early years of their operations, was also developing and strengthening its own systems as they delivered financial support to recipient countries with variable monetary and administrative mechanisms.

This scenario may have prompted one of the stakeholders spearheading the TNVS within the ITN cell to remark as follows:

"...GFATM have not always committed themselves to the development process. They call themselves development agencies, they do not want to hear about the context in which their money is being spent...they had no understanding or compassion that there are health systems constraints that one cannot solve within a 5 year programme (16^*th *^January 2006)."

Most stakeholders interviewed in this synthesis noted that it would be prudent for GFATM grants in the future to allow a preparatory period within the proposal before the actual start date for implementation. Hence, the preparatory period would include all administrative issues and any other unforeseen logistical issues, which the recipient country would put in place prior to the implementation of the grant.

### Bottleneck one: procurement, tendering procedures and structures for implementation

The TNVS was one of the first proposals to be accepted worldwide by GFATM in the first round for the call of proposals in 2002. In accordance to one stakeholder, *"...one of the first ones around the world and one of the best ones too...(29^*th *^October 2006)." *But in spite of this, taking off and becoming active was another issue.

In the Tanzanian context, the TNVS grant took one year from 2002–2003 for tendering of contractors who would be involved in the actual implementation of the programme. These included the Mennonite Economic Development Agency, the World Vision International, the Ifakara Health Research and Development Centre and the London School of Hygiene and Tropical Medicine (LSHTM). The time was also spent in appointing a team leader for the programme. In 2003, the team leader was recruited at the beginning of the year and an administrator and a communication officer were recruited at the end of 2003. The recruitment of the technical staff for the ITN cell was subjected to the normal procurement procedures of the MoH through competitive bidding. The process involved, preparations of the expression of interest through advertising of the posts, calling for the proposals, evaluation, awarding and negotiations. Financial support for the technical staff of the ITN cell was made available from the Swiss Development Corporation.

### Bottleneck two: principles of handling government foreign currency

The other bottleneck that was faced by the MoH, after being awarded the TNVS agreement, included principles of handling foreign currency by the government as stipulated by the MoF. The MoF referred to an Act of the government, which states that foreign money from donors availing financial support to the United Republic of Tanzania through the public sector should be channelled through the MoF before being handled by the recipient ministry concerned. The MoF was the sole custodian of that fund and would be the one signing the grants on behalf of the Government of Tanzania rather than the implementing ministry. This was in spite of the fact that in the TNVS grant proposal, the Principle Recipient (PR) was the MoH. In this respect, the PR had been selected by the Country Coordinating Mechanism in the country to be legally responsible for programme results and financial accountability, as stipulated in the GFATM fiduciary arrangements at the time [58]. These organizational issues between the two ministries delayed the signing of the agreement by two weeks from the day it was awarded. A related financial bottleneck was the issue of the sub-contractors identified to implement the TNVS being required by government procedures to pay VAT. However, the GFATM had set a condition in their guidelines for the application for grants at the time, that the grants that they would award were not to be used by the recipient country to pay VAT[59]. One of the stakeholders, who had been selected independently as the 'Local Fund Agent' of the GFATM grants in the country was Price Waterhouse Coopers. As the Local Fund Agent, they monitored the implementation of the activities of the GFATM, including the contracting negotiations and agreeing on the provisos and verifying the plans at the initial stages [58]. Price Waterhouse Coopers worked tirelessly with the MoF to get a government notice to be signed by the MoF, which was subsequently placed in the government gazette, to relieve the recipients of the GFATM award on the payment of VAT.

The Local Fund Agent is only a medium supporter to the ITN subsidy scheme in the key steps towards the implementation of the scheme, as he confronts the complexities of the procurement hurdles of the Central Tender Board and MoF bottlenecks (Figure 2). His influence over the scheme however shifts to 'medium/high' as he puts all his efforts to ensure that there are solutions to overcome the problems (Figure 6).

Adding the bottlenecks mentioned above equalled to a whole year's delay in the implementation of the TNVS programme. In fact, one of the respondents was quoted as follows, "*The GFATM was close to scrapping the programme. It took a very senior official from the GFATM to come to Tanzania and institute negotiations so that the programme was given a new life. Basically by saying that the start date is different from the actual start date that was originally in the contract *... (29^th ^October 2006).' Furthermore another interviewee, who was an International Public Health specialist advising the MoH on health issues, commented, *"...by coincidence I was in Tanzania when the Portfolio Manager was in Tanzania definitely with instructions to stop the project because of an 11 months delay in issuing contracts. They felt that this was due to technical incompetence (18^*th *^October 2006)."*

The GFATM at the international level hailed Tanzania for being one of the first countries to be awarded a GFATM grant and were confident that they would not be disappointed in the implementation of the intervention. However, within nine months of the release of the funds to the MoH, and no implementation having taken place, they quickly reverted to being 'opposed,' to the intervention short of scrapping the grant as observed in Figure 2.

### Joint efforts open a window of opportunity in salvaging the GFATM grant

A joint memo was written by the Chair, Roll Back Malaria 'Working Group on scaling up ITNs' and the RBM Secretariat Focal Person, East African Regional Network to the GFATM, in August 2004 requesting for a 'No-cost Extension of Phase I: Jan 2005 – 31^st ^October 2005 [60]'. This memo was copied to the Permanent Secretary of the MoH, the Executive Secretary of RBM Secretariat and WHO.

In part the request is quoted as follows *"...Despite the delays, much has been accomplished that is essential to a rapid and efficient roll out of the programme. In the 18 months since January 2003, there have been dramatic increases in domestic commercial market distribution and sales of both bundled and treatment kits (2.3 million ITNs) and separate Insecticide Re-treatment Kits (2.6 million kits) for existing nets even without the voucher due to strong demand creation supported by the NATNETs programme and the low cost of nets on the domestic market. The voucher will now come at the best time to add greater rural penetration and equity of access to the rapidly growing culture of ITNs...The preparatory stage also included a pilot voucher distribution in two districts to be completed and lessons incorporated into the first design of roll out.... Counterfeit resistant vouchers have been designed, produced and procured...detailed work plans for a staggered nation wide launch schedule have been developed... *[60]."

The memorandum also stressed that there was no blue print elsewhere for a country-wide discount voucher scheme, earmarked for the control of malaria. Secondly, it used a novel approach of bringing together and building two existing systems: the public sector arm, where the pregnant women were receiving the discount vouchers, and the private sector distribution of the commodity, in a new configuration in order to achieve high coverage, efficiency, equity and sustainability of ITN use by the most vulnerable group.

One interviewee remarked as follows in relation to the uniqueness of the scheme, *"This is not only the first voucher project done in Tanzania. It is the first nation-wide voucher programme addressing health that has ever been done in Africa (2^*nd *^February 2006)."*

The memorandum also noted that changes in public systems and strengthening take both time and careful preparation to get right. However once accomplished provide a solid foundation for rapid and sustained progress [60].

The GFATM endorsed this request and subsequently the NATNET project lost almost 2.5 million USD due to the delay.

In relation to the reaction of the GFATM to the delays that Tanzania experienced, one stakeholder had the following point of view....*"the GFATM as a funding agent, for them it was expedient to fund projects that bought nets hence less logistics as all the money would be spent in the first week. As opposed to a systems approach which was intended to spend money continuously over time....they would prefer campaign type of approaches rather than system strengthening approaches (18*^*th *^*October 2006)."*

Joint efforts between national and international stakeholders opened a window of opportunity in salvaging the GFATM award for the implementation of the TNVS.

### Contractors of the Tanzania National Voucher scheme and the challenges they faced

The three major contractors in the implementation of the TNVS had their own experience and challenges as they were learning-by-doing in this massive scale-up exercise of ITNs in the country. In order to have a sense of how the early days of implementation faired and their own experiences they were included in the stakeholders analysis.

The logistic contractor was MEDA, responsible for all the logistic arrangements for the production of vouchers, identifying the retailers and wholesalers and reimbursement of the manufacturers of the mosquito nets. The Director of MEDA at the time described their responsibilities as the logistic contractor in the following manner *"....designing and printing of vouchers, ensuring vouchers get to the District Medical Officer and then to the Reproductive and Child Health clinics, identifying and signing up the retailers and wholesalers and the net manufacturers through out the country....We are involved in every step of the movement of the voucher including the final step which is the payment to the wholesalers and the manufacturers (2*^*nd *^*February 2006)."*

Regional managers from MEDA were placed in each region. They approached the district on the implementation of the TNVS through the District Medical Officer (DMO). World Vision International and Care International were appointed as the training and promotion contractors. Their responsibilities included designing promotional materials for TNVS. Apart from training the Council Health Management Teams and the RCH staff, they also included the District Planning Officer, the District Executive Director and the District Cultural Officer of the respective district. Ifakara Health and Research Development Centre and the LSHTM were the monitoring and evaluation contractors for TNVS. The institutions were independent in their monitoring with a schedule that included different types of surveys. These were household and health facility surveys, qualitative studies through in-depth interviews, tracking of vouchers and retail auditing. The baseline for the household survey was carried out in July-August 2005 and it has been repeated yearly thereafter [22].

The Director of MEDA expressed the fears that he had prior to the implementation of the TNVS programme, especially bearing in mind there was no blue print to follow. He was quoted as follows *'....fear of the voucher being produced as forgery, how the clinic staff would react to additional responsibility, how would the retailers and wholesalers perceive vouchers?.........some of the wholesalers said that if it is the government we do not want to be involved. An NGO we can be involved (2*^*nd *^*February 2006).'*

When probed as to why the private sector was weary of working with the government, the Director explained *"....There is a lot of suspicion in the private sector about the government and actually vice versa because one of the things that you struggle with is that many people in the government and Non-Governmental Organizations see business as evil. It is very difficult to see them as potential partners......... The reality is this project does not work without the public sector support at all or the private sector support at all and really the private sector has reacted even better than we thought they would. You have manufacturing companies which have purchased 5 or 6 trucks, because the distribution is so much more developed (2*^*nd *^*February 2006)."*

Another challenge observed was the underestimation of the vastness and geographical complexity of Tanzania. The topography in some districts really challenged them where districts had health facilities built in complex infrastructure. The Manager of the Logistics Component in MEDA also mentioned the problem of existing revolving fund projects within some of the health facilities in the country. The MOH had taken action on this issue by writing a memorandum to all DMO's informing them that given the nature of the voucher scheme, it was deemed essential that existing ITN activities do not contradict or hinder the principle of PPPs, which the TNVS was embarking upon. The regions were requested to examine ways in which existing ITN activities (such as revolving funds, sale of nets by health facilities, and free distribution of ITNs) could be phased out once the TNVS was introduced in the respective districts. Furthermore the districts could explore means of integrating the discount voucher scheme so as not to create two parallel systems, which would be difficult to implement.

The training and promotion contractor alluded to the timelines for the implementation of the TNVS. He noted that the period of covering the whole country with the scheme was too short. He took into cognizance the year that was spent on administrative and logistic issues, after the signing of the grant in 2002 up to the actual launch in 2004 as already mentioned. The contractor felt that the work was very intensive. It was also noted by this contractor that some retailers, which had been selected in respective districts to sell ITNs to the pregnant women, had a tendency to inflate the prices of the mosquito nets. Hence, this became a barrier to the pregnant woman accessing the mosquito net against the intended policy of the intervention.

The monitoring and evaluation contractor noted several challenges in two surveys, which had been conducted at the time of this synthesis. In a qualitative study conducted in 2006 in four districts by the monitoring and evaluation contractor, the nurses expressed that there was work overload since the setting in of the voucher scheme. It was also apparent that there was a period where the districts experienced voucher stock outs, which were related to administrative arrangements of replenishment by the CHMTs. As noted by the other contractors, the RCH staff sometimes became a barrier to women as they demanded a proof of 'top-up.' Another problem observed was that the vouchers were not reaching the remotest part of the districts, as they were not carried by health workers who went to outreach and mobile services within respective districts. The contractor also noted that some of the women interviewed were not aware of the 'face-value' of the voucher. They equated the voucher entitling them to a cheap net and did not perceive that they had to pay a 'top-up.' The coordinator of the monitoring and evaluation component of TNVS expressed that the experience the IHRDC had on social marketing of ITNs in Kilombero-Ulanga project in 2000–2002 was very useful in the large scale country wide discount voucher scheme [19].

### The subsidy scheme is challenged with a directive from the Head of State

Another challenge that faced the subsidy scheme was a directive from the head of state of the URT related to free delivery of mosquito nets in the country. The World Economic Forum, a not-for-profit organization which holds annual meetings on various topics, held its summit in Davos, Switzerland with the theme 'Taking Responsibility for Tough Choices [61]'. One of the agendas put forward at the meeting was debt relief for Africa. Professor Jeffrey Sachs, Director of the Earth Institute at the University of Columbia and Director of the United Nation Millennium Project, mentioned at the meeting that debt relief was the only hope for economic development in Africa. He called for the cancellation of multilateral debt to the most impoverished nations in Africa.

The honourable President of the United Republic of Tanzania, Benjamin William Mkapa was at the time one of the Commissioners of the Commission of Africa at the WEF. In response to Dr Sachs remarks, he made the following statement, *" Each year I must find several million dollars to service the Tanzanian debt. I can use that money for bed nets, which would prevent the loss of 1 million lives every year to malaria."*

In response to the honourable President's plea, a famous actress – Sharon Stone – pledged USD 10,000 to buy bed nets for Africa. She wanted to raise 1 million USD, which would go directly to the GFATM to buy mosquito nets to stop deaths from malaria in the developing world [61].

In March 2005, the Tanzania National Coordinating Mechanism [62] of the GFTAM met to negotiate on the best use of the 'Sharon Stone' funds (Alex Mwita, personal communication). After a lot of debate and in spite of the MoH's position in relation to the delivery of mosquito nets through PPPs, it was agreed that LLINs would be bought and delivered free-of-charge with the measles, Vitamin A and de-worming campaign, which was to take place country-wide in July 2005. The target regions were Lindi and Mtwara as they had the highest infant mortality (129 and 126 per 1,000 live births, respectively in Lindi and Mtwara) and the highest under five mortality in the country (217 and 212 per 1,000 lives birth, respectively in Lindi and Mtwara), compared to a national average 99 per 1,000 live births for infant mortality and an under five mortality of 112 per 1,000 live births [63].

However, by May 2005 only 20% of the pledge from the World Economic Forum had come through to Tanzania. The MoH had ordered LLINs in advance through the A-to-Z Textile factory, but had to cancel. UNICEF intervened to save the situation by agreeing to bridge the gap. UNICEF readily accepted to take this position as part of their cooperate agenda in protecting vulnerable groups in malaria-endemic areas in Africa through accelerated deployment of ITNs [64].

Conventional nets bundled with insecticide re-treatment kits were ordered from the same textile company and distributed to Lindi in July/August 2005 and Mtwara in December 2005 in the country-wide immunization campaign. A MoH official responded as follows about the free net distribution campaign *"The private sector thought that we are killing the net market in those regions, we are working against the public-private partnerships. But we allayed their fears in that this was a one-off activity not continuous. An offer, a Presidential initiative which we could not go against, the market for nets is not limited to under fives only. The population of under-fives is 20%. 80% of the people still need the nets (3*^*rd *^*February 2006)"*. One of the net manufacturers went as far as writing a memorandum to the Roll Back Malaria Secretariat in Geneva related to the intention of the country intending to distribute ITNs freely to vulnerable groups against the standing agreement that Tanzania had in delivering ITNs through a PPP mechanism (memorandum dated 15^th ^June 2005). In response, the RBM Director stressed that the government had through consultations embarked on the campaign as a *one-off presidential campaign *consequent to the precedents set at the Davos meeting in January 2005. Secondly, that the campaign had not changed the policy of the MoH with regards to the PPP approach in delivering ITNs. Thirdly, she stressed that the Demographic and Health Survey (DHS) results (2004/2005) were evidence that the country had increased ITN coverage in both vulnerable groups compared to the 2000–2001 data. Finally, she alluded to some of the indicators which were being observed from the launched discount voucher scheme, including high redemption rates by pregnant women up to 85% at the time, early attendance of pregnant women to antenatal clinics and an expanding domestic commercial market of bundled mosquito nets through strong demand creation pump-primed by social marketing. An administrator from the ITN cell had the following to say in relation to free ITN distribution *"It was a fact that this letter the private sector wrote went to the Presidential level. The minister had to call us and the Programme manager, myself and the Permanent Secretary. We had to narrate how we reached this consensus, showing all the correspondence....... (24*^*th *^*January 2006)."*

In a meeting held by the NMCP to discuss the possibility of developing an equity voucher to address the 15% of pregnant women who were not redeeming their vouchers for a net, the MoH decided to clarify the issues surrounding free net delivery of mosquito nets in two regions in the country in 2005. The programme manager for malaria read a statement of the Ministry of Health and Social Welfare with regard to ITNs on behalf of the Director of Preventive Services in the MoH [65]. "In summary, the MoH reiterated that the free net distribution in Lindi and Mtwara was an exceptional activity arising from the peculiar circumstances and in no way represented a policy shift within government. The statement also stressed that equitable protection of the most physiologically vulnerable groups will be encouraged through the consideration and development of targeted subsidies designed to encourage commercial sector participation in ITN distribution. Subsidies will be linked to utilization of other essential health intervention packages, such as antenatal and Expanded Programme for Immunization (EPI) services [65]."

### Challenges of the TNVS at the community level

A retrospective cohort of women of child-bearing age from Ikwiriri and Kibiti divisions of the Rufiji district, Coast region, under the Rufiji DSS database were followed up twice in the survey coinciding with the normal demographic surveillance updates. The survey enabled the analyst to collect information on the knowledge of the women on the TNVS, access to ITNs, barriers to access and redemption mechanisms. The manner in which the women solved the challenges they faced in acquiring mosquito nets was also documented.

The cohort of women of child-bearing was matched with the socio-economic status of the households from the Rufiji DSS in 2000. From the 1,048 women who were being followed up, 698 matched in the first survey (February to May 2005) and 678 women matched in the second survey (June to September 2005). It was observed that the number of women in the two surveys did not match due to the specific questions that the survey was asking, including the acquisition of vouchers from the Antenatal Clinic by pregnant women for purchasing a mosquito net, and the observation that there could have been some migration of households from the study site within the five years difference between the two surveys.

### Overall use of mosquito nets in the house holds

The first follow-up of the cohort of women of child-bearing age (referred to as 'study population' hereafter) was during the February to May 2005 survey, which serves as the baseline where the voucher was launched in Rufiji district. The actual date of the launch of the voucher scheme was February 2005. The overall net ownership at household level was 41.8% (292/698) at baseline. In the follow-up survey after the voucher had been in place for eight months, the household coverage was 56.05% (380/678) as shown in Table 3. These results were significant with a higher proportion of households owning mosquito nets in the second cross-sectional survey compared to the first one (p < 0.001).

**Table 3:** Overall ownership of mosquito nets (%) in the households in the 1^st ^and 2^nd ^survey of the study population in Rufiji DSS

Use of Net	Feb -May 05	June – Sept 05	Total
Yes	292 (41.83)	380 (56.05)	672

No	406 (58.17)	298 (43.95)	704

*Total*	*698 (100)*	*678 (100)*	*1,376*

P value is < 0.001 and CI (-19.45, -08.99)

### Proportion of households per wealth quintile in the study population

**Figure 8:**
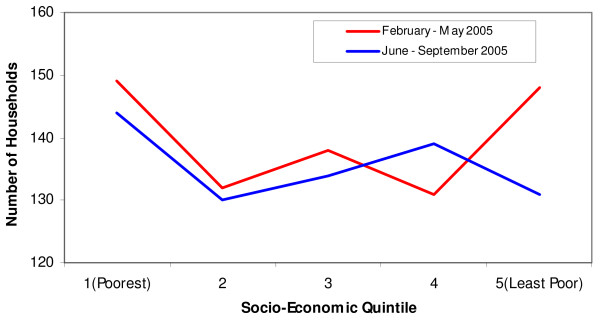
**Number of households per socio-economic status for the 1st (February – May 2005) and 2nd survey (June – September 2005) in the Rufiji DSS**.

The study population was equally distributed in the wealth quintiles in both surveys as shown in Table 4, before matching the study population with ownership of mosquito nets. Figure 8 shows the frequency of households per wealth quintiles in the study population of both surveys.

**Table 4:** Proportion (%) of households per wealth quintiles for the 1^st ^(February – May 2005) and 2^nd ^survey (June – September 2005) in the Rufiji DSS

Quintile	February – May 2005		June – September 2005	
1(*Poorest*)	149	21.35	144	21.24

2	132	18.91	130	19.17

3	138	19.77	134	19.76

4	131	18.77	139	20.50

5 (*Least poor*)	148	21.20	131	19.32

	698	100	678	100.00

**Figure 9:**
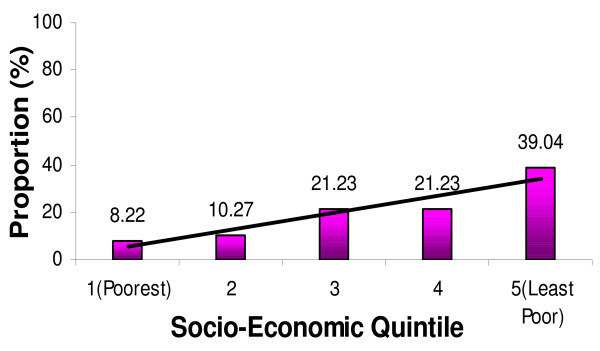
**Ownership of mosquito nets by wealth quintiles for the study population in the 1st survey (February – May 2005)**.

**Figure 10:**
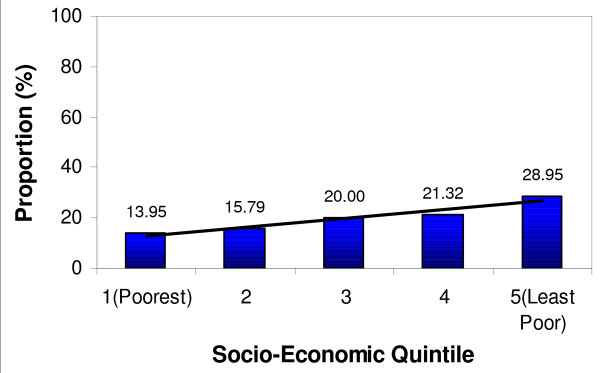
**Ownership of mosquito net possession of the study population per wealth quintile in the 2nd survey – June – September 2005**.

Table 5 shows the ownership of mosquito nets (treated or untreated) by wealth quintiles for the first and second surveys of the study population. There is a significant increase in mosquito net ownership in the most-poor quintile, followed by the second quintile. However, there is no significant increase for mosquito net ownership in the least poor quintile and quintiles 3 and 4. The equity ratio was significant in the second survey (0.48) compared to the first survey (0.21). When the ownership for mosquito nets is disaggregated by wealth quintiles, it is observed that the first and second quintiles have a higher proportion of mosquito nets. The reasons could be that they have had access to the many opportunities around them to get mosquito nets, including the pregnant women voucher and free nets distributed through some missionary hospitals at the time of survey. It is most likely that the least poor did not take the opportunity to acquire more nets, as they already possessed nets in their households. The equity ratio confirms these findings, together with Figure 9, which shows ownership of mosquito nets by wealth quintiles for the study population in the first survey (February to May 2005) and Figure 10, which shows ownership of mosquito nets by wealth quintiles for the study population in the second survey (June to September 2005).

**Table 5:** Ownership of mosquito nets by wealth quintiles for the 1^st ^survey (February – May 2005) and 2^nd ^survey (June – September 2005) of the study population in Rufiji DSS

Yes					No			
**Quintile**	**survey**	**%**	**survey**	**%**	**survey**	**%**	**survey**	**%**

1	24	8.22	53	13.95	125	30.79	91	30.54

2	30	10.27	60	15.79	102	25.12	70	23.49

3	62	21.23	76	20.00	76	18.72	58	19.46

4	62	21.23	76	20.00	76	18.72	58	19.46

5	114	39.04	110	28.95	34	8.37	21	7.05

*Total*	*292*		*380*		*406*		*298*	

### Households with mosquito nets obtained through the voucher scheme

In the baseline survey between February and May 2005 the number of pregnant women who received their mosquito nets through the voucher scheme were 79.44% (228/287), while eight months into the voucher scheme 71.17% (274/385) pregnant women received their nets through this mechanism (Table 6). There was a significantly lower number of pregnant women acquiring their mosquito nets through the voucher scheme in the second cycle. The reason may be due to other sources of mosquito nets, which were available at the time of the survey through the missionary hospitals. The missionary hospital at Mchukwi and some missionary health centres were giving out free mosquito nets to pregnant women who came to deliver at the hospital. This was done to encourage the women to deliver at health facilities rather than at home. The missionary hospitals had been doing this since 2004 (Matron Mchukwi hospital, personal communication). Furthermore free nets were also distributed in July 2005 in a national campaign for measles.

**Table 6:** Proportion (%) of households with mosquito nets obtained through the voucher program from the study population

Voucher	February – May		June – September		Total
		
	2005		2005		
Yes	228	79.44	274	71.17	502

No	59	20.56	111	28.83	170

*Total*	287		385		672

P value = 0.0074 with 95% CI (1.76, 14.78)

### Acquisition of mosquito nets through the Tanzania National Voucher scheme by study population by wealth quintile

**Figure 11:**
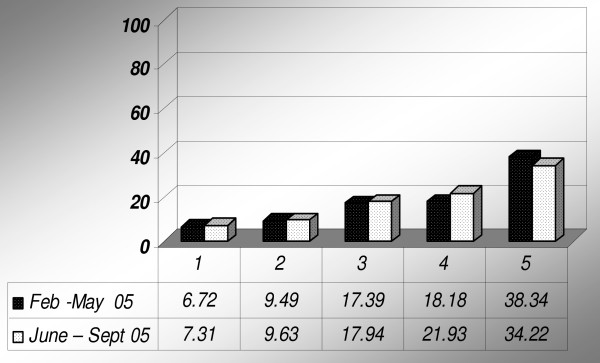
**Proportion of the study population with mosquito nets obtained through the voucher program against their wealth quintiles in the 1st survey (February – May 2005) and 2nd survey (June – September 2005)**.

Figure 11 shows the proportion of the study population with mosquito nets obtained through the voucher programme against their social economic status. In both surveys, the least poor were most likely to purchase a mosquito net through the voucher scheme, at 38% and 34%, respectively. There was no significant difference between the two surveys for the most poor who remained with very low acquisition of mosquito nets in both surveys, at 6.7% and 7.3%, respectively. These results are the reverse of what was observed in the general pattern of household possession of mosquito nets in Table 3. The general pattern in the households was that in the second survey there was a significant difference in the acquisition of mosquito nets in this group compared to the first survey. The reason for the difference observed in acquisition of mosquito nets through the voucher scheme is that in spite of the two surveys not differing in overall net acquisition through the voucher scheme the equity ratio is also very low at 0.21 in the second survey compared to 0.17 in the first survey confirming there had been no improvement of getting nets through this method. The data may be too small to detect this difference at this very early stage of voucher scheme implementation at the Rufiji DSS, but it is also possible that the information about the scheme had not reached a greater proportion of the households at the time.

### Results of focus group discussions

Two Focus Group Discussions (FGDs) were conducted during the course of the survey. The first one was carried out in Ikwiriri Division at Tanga-mbili sub-village (Figure 1). This included 10 women of child-bearing age: with an age range of 18 to 30 years and an average of 25.4 years. Fifty percent of these women were pregnant at the time of the survey and the remaining had infants. The second FGD was carried out in Kibiti Division at Kibiti Central sub-village (Figure 1). Twelve women of child-bearing age were involved, with an age range of 17 to 45 years and an average of 27 years.

Sixty-eight percent of the women had received no formal education. Five percent had dropped out of school before completing their formal primary education. Only 28% had completed their primary education. Sixty-four percent of the women were small-scale farmers in rice paddies, 27% were housewives, and 9% engaged in small business such as selling fish and tailoring.

Malaria was identified as the major public health problem followed by diarrhoeal diseases and lastly HIV/AIDS, by a majority of the women. All the women affirmed that mosquito nets were readily available from the shops, shifting markets and vendors. They quoted that the price range depended on the size of the net: 3,500 Tshs – 4,000Tshs (about 3.5 – 4.00 USD at the time of the survey). They affirmed that the nets were bundled with an insecticide re-treatment kit. On the question of knowledge of the discount voucher scheme, one or two members of the groups described the scheme and others confirmed. Only two women in both FGDs were able to give the correct value of the voucher. The responses ranged from 350 Tshs – 2,250 Tshs. It was evident, due to the low literacy level of the women, that they did not actually comprehend what the face value of the voucher was. On the other hand it also reflects the level of sensitization that they had received from the RCH Coordinators when they attended their antenatal clinic and were first introduced to the scheme.

In addition to receiving vouchers from the public health facilities and the mission hospital, four mothers mentioned that they had received free mosquito nets from one of the mission health centres. However, 50% of the women said that they had received free mosquito nets during the measles campaign held in July 2005. In this campaign, all children under five received free nets and all of them were bundled with insecticide re-treatment kits. With regards to the problems surrounding the operationalization of the voucher scheme at the household level, two issues were described by the FGDs. One respondent mentioned that the RCH coordinators themselves were not conversant enough with the system hence they caused some one to leave the ANC without receiving a voucher. Even when some pregnant women deliver in the same clinic that they have been attending antenatal check-ups, they still did not get their voucher. Another woman actually confirmed that she had attended antenatal clinic at least twice in her pregnancy and up until the time she had delivered, she was never given a voucher. The second problem cited was the hiking of prices of mosquito nets by some of the shop-keepers since the discount voucher scheme began. Thus, the shop-keepers were actually benefiting from the discount voucher by over-pricing the mosquito nets compared to the price before the scheme began.

Interviews were also conducted with RCH Coordinators from three health centres and one hospital, which were randomly selected within the Rufiji DSS. The health facilities were the Ikwiriri Mission, (a dispensary), the Kibiti Health Centre (a public facility), Mariamu Consolota dispensary (a mission dispensary) and Mchukwi Hospital (a missionary hospital) (Figure 1).

The interviewees confirmed that the voucher scheme begun on the 14^th ^February 2005 in two facilities and on the 15^th ^February 2005 in the others. They received training for one day on issues related to malaria and the discount voucher scheme. The trainees were from World Vision International and the District Nursing Officer (she is the Integrated Management and Childhood Illness (IMCI)/Malaria focal point in the district). The RCH coordinators pointed to three major problems which they had observed since the scheme begun: 1) voucher books could be depleted before they were replenished by the district, 2) there were women who could not afford the top up to the voucher and, hence, did not redeem the voucher for a mosquito net, and 3) there were women who actually move into the paddy farms during the farming season and slept in fragile houses and usually these families were extremely poor. There was a very small probability of these women accessing the voucher let alone redeeming it.

### Analytical features of the challenges faced in the implementation of the TNVS

The Abuja declaration clearly stipulated that countries endemic for malaria were obliged to ensure that there was a reduction of taxes and tariffs for all mosquito net products so that they became affordable to the communities [66]. To meet this challenge, early in 1998, the MoH held a meeting with other relevant government sectors, multilaterals, bilaterals, the private sector for – profit and non-profit to set-up strategies to tackle the issue of taxes and tariffs on mosquito net products in Tanzania. The strategies included lobbying members of parliament and influential individuals, wide dissemination of the outcome of the meeting to international organizations and the media. The results were fruitful and taxes and tariffs on mosquito net products including insecticides used to treat the nets were reduced. His Excellency Benjamin William Mkapa at a fund raising dinner held in 2000, toward the 'Comprehensive malaria control project in Zanzibar' had this to say on the issue of taxes "...*Tanzania was the first country to take action on the so-called "malaria taxes" last year, by reducing the total taxes on mosquito nets and insecticides to fight malaria to a combined rate of 5% only. This has made mosquito nets affordable to the people*... [67]". Many countries at the time had not rationalized their taxes and tariffs on mosquito net products. To date only 15 of the 39 Abuja signatories of the malaria declaration in 2000 have reduced taxes and tariffs on this life-saving intervention [68].

The MoH was strategic in their approach to the tax rationalization agenda in (1) allowing a broad discussion around the tax reform required through a multisectoral meeting, (2) lobbying key national individuals and organizations, (3) strengthening alliances with relevant international organizations such as the World Trade Organization, WHO and the World Bank, and (4) making use of the media to bring the issue to the forefront in the eyes of the public. Most importantly the process was owned and led by the government itself. Ironically, the situation lasted only for two years: while the MoH was struggling to ensure how best the mosquito nets could now be delivered to the communities, the MoF re-introduced taxes and tariffs on mosquito net products in the country. The reason behind this is the generation of internal revenue through raising the tax base of some commodities [69]. The synthesis could not explicitly identify whether the government officials from the MoF which were involved in the re-introduction of the taxes were different from those that reduced the taxes in 1999/2000; what is evident is that an overarching agenda of raising the domestic revenue of the country is overshadowing the efforts of the MoH in the control of malaria. All civil servants are governed in terms of their loyalties, values and expertise and avidly follow through their agenda in many instances. The MOF officials in this context have disregarded the history of the removal of taxes on mosquito net products in the first place having had no consultations with other relevant sectors on the subject, where they would have gleaned the repercussion of their decision on health [54].

Evidently, the rationalization of taxes and tariffs by the MOH under the particular clause that affected the mosquito nets also had an effect on the MSD, who independently struggled with the new tax bill. Subsequently, lobbying was carried out by the NMCP and key stakeholders and, in 2004, within the new fiscal budget of the government taxes and tariffs on mosquito net nets and related products were reduced again. The MoH, through its steering committee for NATNETS, was able to create an appropriate space to manoeuvre, in a complex policy issue and engaged other actors on the scene in a timely manner to further the malaria control agenda.

Tanzania was one of the first countries to receive a GFATM award for malaria control. Nonetheless, it took the country almost one year from the signing of the grant to the implementation of the discount voucher activities. The hurdles faced by the MoH during this period was partly due to the procurement bottlenecks within the Central Tender Board, but was also related to the organizational arrangements of the GFATM at the time: (1) the GFATM was a newly-formed funding organization for the three diseases. It was challenged with an urgent need to show results in the recipient countries and balance this with the latter managing the new financing mechanism [70], (2) at the international level it had high staff turnover with different portfolio managers assigned to countries within less than a year hence reducing the institutional capacity of these managers to follow-up on the design and scope of the proposals, (3) GFATM needed to be sensitive to the contextual and systemic issues surrounding recipient countries in as much as they demanded performance based management [71], and (4) the organization had to make provision for recipient countries to build health systems through the grants they disbursed which are critical for successful programmatic implementation. These include strengthening the human resource capacity, contracting and procurement procedures, concrete monitoring and evaluation plans etc. One of the major challenges alluded to by the WHO in reaching the Millennium Development Goals is failing or inadequate health systems. Without building and strengthening these systems it will be difficult to reach communities with the desired health outcomes on time, reliably and in sufficient quantities [72]. It is encouraging that the GFATM has since 2005 incorporated health systems strengthening issues within country proposals [73].

The international community through the RBM working group on scaling up ITNs made a historical and timely intervention to salvage the discount voucher programme when it was threatened to be 'scrapped' by the Board of the GFATM in 2004. Their sensitivity to the public health systems in the country enabled them to articulate the nature of the unique approach that Tanzania had embarked upon in the delivery of ITNs through a PPP to the GFATM and request for a no-cost extension so that the programme could continue.

In 2005, a year into the implementation of the TNVS and at a time when the NMCP had covered 75% of the country with the scheme; a presidential initiative brought uncertainty to the status quo of the PPP initiative amongst stakeholders. Indeed, the fact that His Excellency the President of the United Republic of Tanzania recognized malaria as one of his priority agenda's is apparent when he made a plea at the World Economic Forum to channel the savings from debt relief towards the purchase of mosquito nets [61]. The problem was the consultative or non-consultative process that the country pursued in the delivery of the mosquito nets purchased through these funds [74].

A series of instructions were received by the NMCP from the GFATM (who were given the task of managing the funds), in relation to the presidential initiative. The GFATM desired the funds to be used for quick wins, high visibility and high profile. The Executive Director of GFATM at the time suggested saturating an area of high malaria transmission with at least one LLIN per family to demonstrate a big measurable impact [74]. But the country took a different approach. The NMCP was engaged in rapid planning for the funds with the Tanzania National Coordinating Mechanism, who in turn were receiving pressure from the President's office, the Prime Minister's office and the MoH to finalize the proposal for the initiative. Finally a decision was made to deliver free LLINs to two regions with the highest poverty rates and infant mortality.

Notably, the Presidential initiative had turned the malaria agenda into a *crisis event*. The stakes were high, there was high visibility and there was a lot of uneasiness not only for the domestic net manufacturers who had invested heavily in the production of mosquito nets but amidst the members of the NATNETs steering committee as well. The institutions the steering committee members belonged to had availed considerable grants towards the PPP approach in the delivery of nets in the country. One member of the steering committee was quoted as follows '...*we have been mandated by the Ministry of Health to advise the government on matters related to scaling up of ITNs in Tanzania, but in this Presidential initiative, we have been left out...'*. Members of the steering committee, who were mostly from the bilateral organizations such as the UK Department for International Development, the Swiss Development Cooperation, the Development Cooperation for Ireland and the Royal Netherlands Embassy became moderate/poor supporters of the programme in this phase of the implementation of the scheme (Figure 3) and had low influence on the events around them (Figure 6).

As for the net manufacturers, they were really desperate and wrote a memorandum straight to the RBM Secretariat in Geneva reiterating their fears with regards to the agreements that the manufacturers had been enforced with the government on the PPP approach (Figures 3 and 6). The RBM Secretariat responded by carefully outlining that this was a 'one-off' event and the country would not abandon its core strategy in the delivery of mosquito nets.

In the few months that the grant for implementing the President's initiative was being handled by the NMCP, constraints were also observed between the NMCP and UNICEF. In spite of the fact that this multilateral organization had bridged the gap in a very timely manner when the grant funds from the World Economic Forum were delayed, they strongly urged the NMCP to make provision towards the delivery of free nets (Figure 3). However, the MoH was reluctant to change their ITN policy due to a one-off initiative of free delivery of nets in two regions. A number of issues are drawn out through the free delivery of nets in this Presidential initiative: (1) implementation of the policy was in the hands of many different groups and mostly influential figures in the government including the Head of State, (2) there was conflict as the delivery mechanism of nets in the country is changed, (3) the outcome of the conflict was being felt in the public arena, (4) the malaria agenda was turned into a macro-political issue characterized with high stakes, high visibility, in which the government takes complete control in driving the agenda with little or no consultation with other stakeholders, (5) the timing of the initiative was crucial as the funds had to be spent quickly and make a visible impact.

Once all the plans were in place and implementation was underway, the MoH stepped back and called upon its malaria stakeholders to expound the fact that the free net delivery carried out in Lindi was a one-off Presidential initiative, which was beyond their control. They also assured their partners that the mainstay of the Ministry's delivery mechanism was the PPP initiative approach.

The challenges observed by the contractors who were operating the scheme allude to the fact that once policy is formulated, implementation can take on a different angle from the initial goal depending on the bureaucrats at the grass root level [75]. This was reinforced by the focus group discussions where only 60% of the women who were eligible to receive their vouchers actually acquired it. They also underestimated the magnitude of the work and the difficult terrain that features in some districts in the country.

The poverty index in the cohort of women of child-bearing age, which were followed up in the two cycles of the cross-sectional survey, reveal that the most poor are still disadvantaged in redeeming their vouchers for a net. In the monitoring and evaluation household surveys conducted in 2006, it was observed that the least poor women and those living in early launch districts were more likely to have reported receipt of pregnancy voucher than the most poor women and those living in the late launch districts.

A cross-sectional survey carried out in Rufiji in 2005, with a large sample size to observe the contribution of various delivery mechanisms to net use by socio-economic status, was very significant [28]. Net coverage was far higher for the least poor with more than 80% of the better off quintile possessing a net. In the least poor quintile, over half obtained their nets from the commercial market at full price (the most popular source of nets for all other socio-economic groups). Monitoring studies showed that coverage of the three intermediate wealth quintiles was relatively even, coverage of the poorest was consistently and substantially lower for both voucher subsidized and freely distributed nets. Notably the study has also shown that the issue of inequity where subsided mechanisms are used for delivery of goods also applies to goods delivered free of charge. The larger cross-sectional survey study in Rufiji observed that the majority of nets used by infants were obtained through the national voucher scheme and nets provided free to the end user during the child vaccination campaign supported by the Red Cross. Net coverage achieved amongst young children was 50% and over 30% of coverage amongst older children [28]. Clearly the mix of catch-up and keep-up strategies in this very poor rural setting has substantially increased the coverage of mosquito nets in discrete populations of the community in a very complementary manner. The RBM working group on scalable malaria vector control (WIN) underscores that '*for targeted distribution – a single mass distribution catch-up campaign followed up by a continuous delivery system integrated into the ANC and EPI catch-up strategy should rapidly achieve and sustain control coverage levels in excess of 80% without the need of further campaigns... *[76].'

## Conclusion

Tanzania took a bold step in the implementation of a country-wide voucher scheme for the delivery of ITNs through a PPP approach to vulnerable groups. The stakeholders had no 'blue-print' in the venture and had to gain experience as the scheme evolved. The challenges they encountered articulated in this synthesis lend invaluable lessons to malaria endemic countries: (a) the consistency of the stakeholders with a common vision to their desired goals is an important strength in overcoming obstacles, (b) senior politicians will often drive the policy agenda when the policy in question is a 'crisis event', the stakes are high and there is high visibility, (c) national stakeholders in policy making have an advantage in strengthening alliances with international organizations, where the latter can become extremely influential in solving bottlenecks as the need arises, and (d) conflict can be turned into an opportunity as the Presidential initiative has lent Tanzania a learning ground in 'catch-up' campaigns, which had not been carried out at that scale in the country. The outcome is a hybrid of mechanisms including catch-up and keep up in the newly developed National Malaria Control Strategic Plan 2008 – 2013 [77].

The United Republic of Tanzania in congruence with its PPP policy has invested heavily in the scale-up of LLINs in the A-to-Z net manufacturing company in Arusha, where in 2005 it contributed to the building of a new tarmac road to the plant, including the installation of electricity and water. This was to the tune of about 2.5 million USD (Anuj Shah, personal communication). The country has been consistently positive in the PPP approach to ITNs as seen from 2000 when His Excellency the President of the URT, at a fund raising dinner for the comprehensive malaria control project in Zanzibar had this to say *..."I must caution however, that all political will to fight malaria notwithstanding, we can only succeed to meet our goals if there is active participation and support of all stakeholders *[67]."

## Abbreviations

ACT: Artemisinin-based combination therapy; CHMT: Council Health Management Team; ANC: Antenatal clinic; CARE: An International NGO tackling poverty; CHMT: Council Health Management Teams; CSSC: Christian Social Service Commission; DCI: Development Cooperation of Ireland; DFID: Department for International Development; DHS: Demographic Health Survey; DMO: District Medical Officer; DSS: Demographic Surveillance System; EPI: Expanded Programme for Immunization; FGD: Focus Group Discussions; GFATM: Global Fund for AIDS, TB and Malaria; HSR: Health Sector Reform; IDRC: International Development Research Centre; IMCI: Integrated Management of Childhood Illnesses; IRDC: Ifakara Research and Development Centre; ITNs: Insecticide Treated Nets; KINET: Kilombero Net Project; LLINS: Long-lasting insecticide-treated Nets; LSHTM: London School of Hygiene and Tropical Medicine; MDGs: Millennium Development Goals; MEDA: Mennonite Economic Development Association; MIM: Multilateral Initiative for Malaria; MOF: Ministry of Finance; MOH – Ministry of Health; MSD: Medical Stores development; NATNETs: National Insecticide-Treated Net Strategy; NIMR: National Institute for Medical Research; NMCP: National Malaria Control Programme; PMI: President's Malaria Initiative; PPP: Public-Private Partnership; PSI: Population Service International; PWC: PriceWater HouseCoopers; RBM: Roll Back Malaria; RCH: Reproductive and Child Health; RDSS: Rufiji Demographic Surveillance System; RNE: Royal Dutch Embassy; SDC: Swiss Development Cooperation; SMARTNET: Social Marketing of Insecticide Treated Nets; STI: Swiss Tropical Institute; TaNAAM: Tanzania Alliance Against Malaria; TEHIP: Tanzania Essential Health Intervention Project; TNM: Tanzania Net Manufacturers; TNVS: Tanzania national Voucher Scheme; TRA: Tanzania Revenue Authority; TRP: Technical Review panel; UNICEF: United Nation's Children's Education Fund; VAT: Value Added Tax; WB: World Bank; WEF: World Economic Forum; WHO: World Health Organization; WVI: World Vision International

## Competing interests

The authors declare that they have no competing interests.

## Authors' contributions

RJA, DS, LG and took part in the design of the case study. RJA collected, collated and analyzed the data at all levels from the field work to the national level in-depth interviews. LG was instrumental in drafting and guiding the manuscript with regard to the policy implications of our key results and guided the policy analysis in all the phases of the research. DS, EM and RJA were instrumental in designing the field tools and undertaking the field work in the Rufiji Health Demographic Surveillance Site. RJA, DS, LG, EM and FM took part in drafting the manuscript.
